# Controlled biodegradation of magnesium alloy in physiological environment by metal organic framework nanocomposite coatings

**DOI:** 10.1038/s41598-021-87783-x

**Published:** 2021-04-21

**Authors:** Mohammad Amin Khalili, Elnaz Tamjid

**Affiliations:** 1 Department of Biomaterials, Faculty of Biological Sciences, Tarbiat Modares University, P.O. Box 14115-154, Tehran, Iran; 2 Department of Nanobiotechnology, Faculty of Biological Sciences, Tarbiat Modares University, P.O. Box 14115-154, Tehran, Iran

**Keywords:** Biotechnology, Engineering, Materials science

## Abstract

Magnesium-based implants (MBIs) have recently attracted great attention in bone regeneration due to elastic modulus similar to bone. Nevertheless, the degradation rate and hydrogen release of MBIs in the body have to be tackled for practical applications. In the present study, we present a metal–organic framework (MOF) nanoplates to reduce the degradation rate of AZ91 magnesium alloy. Zeolitic imidazolate frameworks (ZIF-8) with a specific surface area of 1789 m^2^ g^−1^ were prepared by solvothermal methods, and after dispersion in a chitosan solution (10% w/w), the suspension was electrospun on the surface of AZ91 alloy. Studying the degradation rate in simulated body fluid (SBF) by electrochemical analysis including potentiodynamic polarization and electrochemical impedance spectroscopy reveals that the degradation rate of the surface-modified implants decreases by ~ 80% as compared with the unmodified specimens. The reduced alkalization of the physiological environment and hydrogen release due to the implant degradation are shown. In vitro studies by fibroblasts and MG63 osteosarcoma cells exhibit improved cell adhesion and viability. The mechanisms behind the improved degradation resistance and enhanced bioactivity are presented and discussed. Surface modification of MBIs by MOF-chitosan coatings is a promising strategy to control the biodegradation of magnesium implants for bone regeneration.

## Introduction

Despite the self-healing ability of bones, severe injuries caused by accidents or fractures require bone grafting^1^. Besides autografts, biocompatible and bioactive scaffolds are required to accelerate the healing process of damaged bone^2^. Recently, biodegradability, biocompatibility, and compatible mechanical properties of magnesium-based alloys with bone have raised significant interest in bone implantation^3^. Magnesium itself is the fourth most abundant element in the body that participates in most of the biological functions^4^, enzymatic reactions^5^, tissue repair^2^, protein and nucleic acid synthesis, mitochondrial activity, plasma membrane robustness, and RNA transcription processes^6,7^. Many studies have shown that porous magnesium scaffolds can easily be degraded at endosomal sites and dramatically stimulate new bone formation and stimulate angiogenesis^5^. Of interest, the implant degradation products enhance osteoblast proliferation, differentiation, and expression of osteoporotic markers, while the excess amount does not cause cytotoxicity in the human body and is generally excreted through the urethra^7^.

Albite all the aforementioned advantages of MBIs, the low corrosion resistance of magnesium alloys in physiological environments (PE) results in a rapid loss of mechanical integrity and growth of hydrogen bubbles that severely impair the bone healing process^8^. Therefore, the bioreaction rate of MBIs in PE must significantly be reduced to facilitate the use of lightweight magnesium alloys for bone implantation. Laboratory studies have estimated the critical tolerance of H_2_ to be less than 0.01 ml/cm^2^ day^9^. So far, different strategies have been employed to reduce the biodegradation rate of BMIs in PE. Alloying and surface modification are the most widely utilized strategies to minimize the complications of magnesium implantation into the human body^10^. It has been shown that non-toxic alloying elements such as Ca, Zn, Sr, and Si improve the mechanical stability and biodegradation rate of MBIs in PE^11^. However, the biocompatibility and degradation rate are still deficient for practical applications and may trigger a series of side effects on surrounding tissues after implantation^12^. Surface modification either through physical methods (morphological adjustment without any change in the components) or chemical techniques (surface functionalization, ion infusion, and coatings) are alternative approaches to reduce the biodegradation rate^13^. Among different methods, sol–gel^14^, electrophoretic deposition (EPD)^15^, and plasma electrolyte oxidation (PEO)^16^ have gained considerable attraction in recent years. Different studies have shown that surface modification by depositing a coating layer not only improves biocompatibility, but also encourages bone ingrowth, osseointegration induction, and mesenchymal stem cell proliferation^17,18^.

Polymer coatings provide several advantages for the surface modification of MBIs. Depositing a thin layer of polymeric materials on the surface acts as a physical barrier to make the biodegradation rate of the substrate compared with the bone regeneration rate of the damaged tissue^19^. The barrier film ought to be mechanically stable and hydrophilic to increase cell adhesion and stimulate bone cell proliferation^3,20^. Sang et al.^21^ have shown that surface modification of Mg–6%Zn–10% Ca_3_(PO_4_)_2_ by chitosan prevent severe corrosion in SBF with a significant reduction in the formation of hydrogen bubbles. Heakal and Bakry^22^ employed spin-coating to deposit a thin chitosan film on AXJ530 alloy. They showed that the biodegradation rate of the magnesium alloy in PBS was significantly retarded. Studies of Tiyyagura et al.^10^ indicated that along with the reduced corrosion rate, the chitosan film promoted the formation of the hydroxyapatite layer on the surface. Panahi et al.^23^ employed electrospinning to prepare polycaprolactone-bioactive glass (BG) fibrous composite coatings on AZ91 alloy. Detailed electrochemical studies have determined that the composite coating significantly decreases the corrosion current through retarding ion and electron transport between the surface and the liquid environment. On the other hand, BG particles enhance bioactivity, cell adhesion, and cell proliferation.

In the present work, we used chitosan/MOF composite coatings for the surface modification of BMIs. To our best knowledge, the role of polymer/MOF composite coatings on the biodegradation rate of MBIs in PE has not been reported yet. We employed biocompatible ZIF-8 nanoplates with a porous crystalline structure that has high aqueous stability and exceptional chemical resistance against polar and nonpolar solvents^24^. Due to very high surface area, ZIF-8 nanoplates have high adsorption capacity, thus making this material a suitable candidate for drug delivery^24,25^, which could provide additional advantages to prepare drug-eluting coatings on MBIs to prevent bacterial infections^26^ or to deliver growth factors on-site^27,28^. Since the coating layer ought to have mechanical integrity and adherence to the surface during the degradation, fibrous chitosan/ZIF-8 MOF coatings were prepared. Chitosan is a well-known biocompatible biopolymer with rapid blood clotting and homeostatic effect^10,29^. Recent studies on BMIs implants have determined that the chitosan-modified implants are resistant to osteomyelitis (bone infection) caused by Staphylococcus aureus along with improved osteogenesis after implantation^30^. In vivo studies have also determined limited inflammatory response in several 8-week-old adult rats^5^. The fibrous structure of the composite film provides a suitable microenvironment for cell attachment and spreading. The nanofibers can also be penetrated by cells to treat or replace biological targets and hinder the degradation of implants in human body during the treatment^31,32^. We studied the effect of the MOF/chitosan composite films on the biodegradation rate by the Tafel polarization test and electrochemical impedance spectroscopy (EIS). The biocompatibility of the surface-modified Mg alloy is demonstrated and the improved biological performance is discussed based on experimentally measured hydrogen gas evolution and changes in the pH of the cell environment. The MOF/chitosan films show promising capacity for surface modification of MBIs for bone regeneration.

## Materials and methods

### Materials

Biomedical grade AZ91 magnesium alloy was obtained from a local market. The chemical composition of the alloy was determined by a flame atomic absorption spectroscopy, as reported in Table 1. Medium molecular weight chitosan (Mn = 80,000 Da) and polyethylene oxide (PEO) with an average molecular weight of 4,000,000 were obtained from Sigma–Aldrich (USA). Zinc nitrate hexahydrate (Zn (NO_3_)_2_·6H_2_O), 2-methylimidazole, dimethyl sulfoxide (DMSO), and SBF were also obtained from the same company. Absolute methanol and acetic acid (50%) were purchased from Merck (Germany). Dulbecco's modified eagle medium (DMEM) was obtained from Gibco (Scotland). All chemicals were used without further purification.

**Table 1:** Chemical composition of commercially available AZ91 alloy used in this study.

Element	Mg	Al	Zn	Mn	Si	Cu	Fe	Ni	Others (each)
Content wt%	balanced	8.70 ± 0.4	0.55 ± 0.08	0.25 ± 0.05	Max. 0.030	Max. 0.02	Max. 0.01	Max. 0.002	< 0.02

### Synthesis of metal–organic frameworks

ZIF-8 MOF particles were synthesized by solvothermal methods. Zn(NO_3_)_2_ and 2-methylimidazole (MIm) with a Zn^2+^/MIm molar ratio of 1:8 were dissolved in methanol by dropwise adding and gentle stirring. After mixing for 60 min, the solution was transferred in a Teflon-lined autoclave and treated for 24 h at 120 °C. The precipitate was separated by centrifugation and washed three times with fresh methanol to remove any unreacted reagents. The resulting product was vacuum dried to attain ZIF-8 MOFs.

### Surface modification of magnesium alloy

The AZ91 slab was cut into small cubes with dimensions 1 mm × 1 mm × 1 mm by electrical discharge machining. Before electrospinning, a surface treatment including grinding with a SiC emery paper (mesh #180) followed by chemical etching in 1 M HNO_3_ for 30 s was performed. As shown elsewhere^33^, this pre-treatment created a rougher surface and improved the adhesion of electrospun fibers to the magnesium surface.

For electrospinning, at first, an aqueous solution of chitosan was prepared by dissolving the polysaccharide (4 wt.%) in 4 mL acetic acid solution (50%) with a pH = 5.1. The mixture was stirred for 24 h at 250 rpm, and then filtered to remove any residuals. To facilitate electrospinning, PEO with a mass ratio of 1:4 to chitosan was added to the polymer solution and stirred overnight. For the preparation of composite suspensions containing ZIF-8 MOFs, the nanoparticles (10 wt.%) were added to the polymer solution, stirred for 2 h, and sonicated for 45 min. A single nozzle electrospinning apparatus (ES1000, FNM, Iran) was used to deposit chitosan and MOF/chitosan composite films on the surface of the magnesium specimens. The processing parameters were determined based on trial and error efforts. The applied voltage was 21 kV. A flow rate of 1 mL/h was utilized. The distance of the syringe to the aluminum rotating drum (2500 rpm) was 10 cm. To attain films with the same thickness of 30 µm, the processing parameters were kept the same for both chitosan and composite coatings.

### Materials characterizations

Scanning electron microscopy (SEM, Philips, XL30) was used to study the size and morphology of MOF particles. For the phase analysis, X-ray diffraction (XRD) was carried out by a Philips PW 1730 diffractometer (Netherlands) which uses Cu-kα radiation (0.178897 nm,40 kV, 30 mA) in 25 °C. Brunauer–Emmett–Teller (A Belsorp mimi BET, Japan) was employed for the surface area analysis. The size and morphology of the fibers, as well as the thickness of the electrospun films on the surface of the magnesium alloy, were studied by SEM. The biodegradation products after immersion in SBF were also analyzed by SEM and XRD. Besides, electron dispersive spectroscopy (EDS) was used for elemental analysis. To determine the effect of surface modification on the hydrophilicity, the sessile drop technique was employed according to ASTM D7334 standard. A small droplet of deionized water was placed on the surface of the samples by micropipette and photographed by a camera. The water contact angle was determined by the ImageJ software.

### Degradation studies

All biodegradation evaluations were carried out in SBF at 37 °C. Potentiodynamic polarization test was carried out by a potentiostat (302N, Metrohm Autolab, Netherlands). A conventional three-electrode configuration including a working electrode (AZ91 alloy), a saturated calomel electrode (control), and a platinum counter electrode was used. Potentiodynamic polarization curves were recorded by changing the voltage in the range of − 2.5 to 0 mV with a rate of 20 mV/s^34^. To establish dynamic equilibrium conditions, the specimens were immersed in SBF for 60 min before testing. To determine the corrosion current (*i*_corr_), the Tafel slopes were determined in the vicinity of the corrosion potential (*E*_*cor*_)^35^ and used in the Stern–Geary relation^36^:

1 $$icorr = \frac{\beta a \cdot \beta c}{{2.303(\beta a + \beta c)Rp}}$$

βa and βc are the anodic and cathodic Tafel slopes, and Rp is the polarisation resistance that was obtained from the electrochemical impedance spectroscopy (EIS) technique. EIS was carried out at a 1 V DC potential (vs. the standard electrode). A sinusoidal potential amplitude (10 mV) in the frequency range of 0.1–105,000 Hz was applied. The acquired EIS data were fitted to an equivalent circuit by employing the ZView software (Scribner Associates Inc., USA).

To determine the rate of hydrogen evolution during biodegradation, cubic specimens with a volume of 1 cm^3^ were inserted in a transparent plastic syringe containing 60 mL SBF. The container was incubated at 37 °C for 15 days. The amount of hydrogen volume was then determined by measuring the volume change according to the procedure explained in Ref.^37^. Briefly, the volume of the released hydrogen was estimated from the below reaction:

2 $${\text{Mg }} + {\text{ 2H}}_{{2}} {\text{O}} \rightleftharpoons {\text{Mg}}^{{{2} + }} + {\text{ 2OH}}^{ - } + {\text{ H}}_{{2}}$$

For the evolution of 1 mL H_2_, we have:

3 $$\frac{{1\;mL\;H_{2} }}{{cm^{2} \,day}} \times \frac{{1\;L\;H_{2} }}{{1000\;mL\;H_{2} }} \times \frac{{1\;mol\;H_{2} }}{{22.4\;L\;H_{2} }} \times \frac{1\;mol\;Mg}{{1\;mol\;H_{2} }} \times \frac{{24.3\;g\;Mg^{2 + } }}{{1\;mol\;Mg^{2 + } }} \times \frac{{cm^{3} \;Mg^{2 + } }}{{1.81\;g\;Mg^{2 + } }} \times \frac{365\;day}{{1\;year}} = 0.218\frac{mm}{{year}}$$

Statistical analysis was carried out by GraphPad Prism with considering *p < 0.05 as significant. Data are expressed as mean ± standard deviation (SD).

### In vitro studies

L929 fibroblast cells were obtained from the National Cell Bank of Iran (Pasteur Institute of Iran, Tehran) All the specimens were sterilized by 70% ethanol for 30 min in a 24 well-plate and dried under a laminar flow biosafety cabinet (KG-A100, Kimia Gene Co., Iran). The specimens with a surface area of 1 × 1 cm^2^ were then placed in a 24-multi well plate and 5 × 10^4^ cells were seeded on each sample. The control was the well plate without the AZ91 sample. After incubation for selected times, 20 μL of 1 mg/mL MTT (3-[4,5-dimethythiazol-2-yl]-2,5-diphenyl-tetrazolium bromide) solution was added to each well and incubated for 4 h at 37 °C. To dissolve the insoluble purple formazan crystals, the medium was replaced by (DMSO). The absorbance in each well was then recorded at 540 nm through an ELISA reader^38^.

For evaluation of cellular adhesion, human osteoblast-like MG-63 cells (the National Cell Bank of Iran, Pasteur Institute of Iran, Tehran) were used. The cells with a density of 5 × 10^4^ were incubated on the specimens in a 5% CO_2_ humidified incubator at 37 °C. After 3 days, the non-adherent cells were washed out by PBS rinsing. The attached cells were then fixed by a fixative solution containing glutaraldehyde and stained in an alcoholic solution for 5 min and dried. Changes in the pH of the medium were measured by a pH meter (pH7110, Inolab, Germany).

## Results

### Characteristics of the metal–organic frameworks

**Figure 1:**
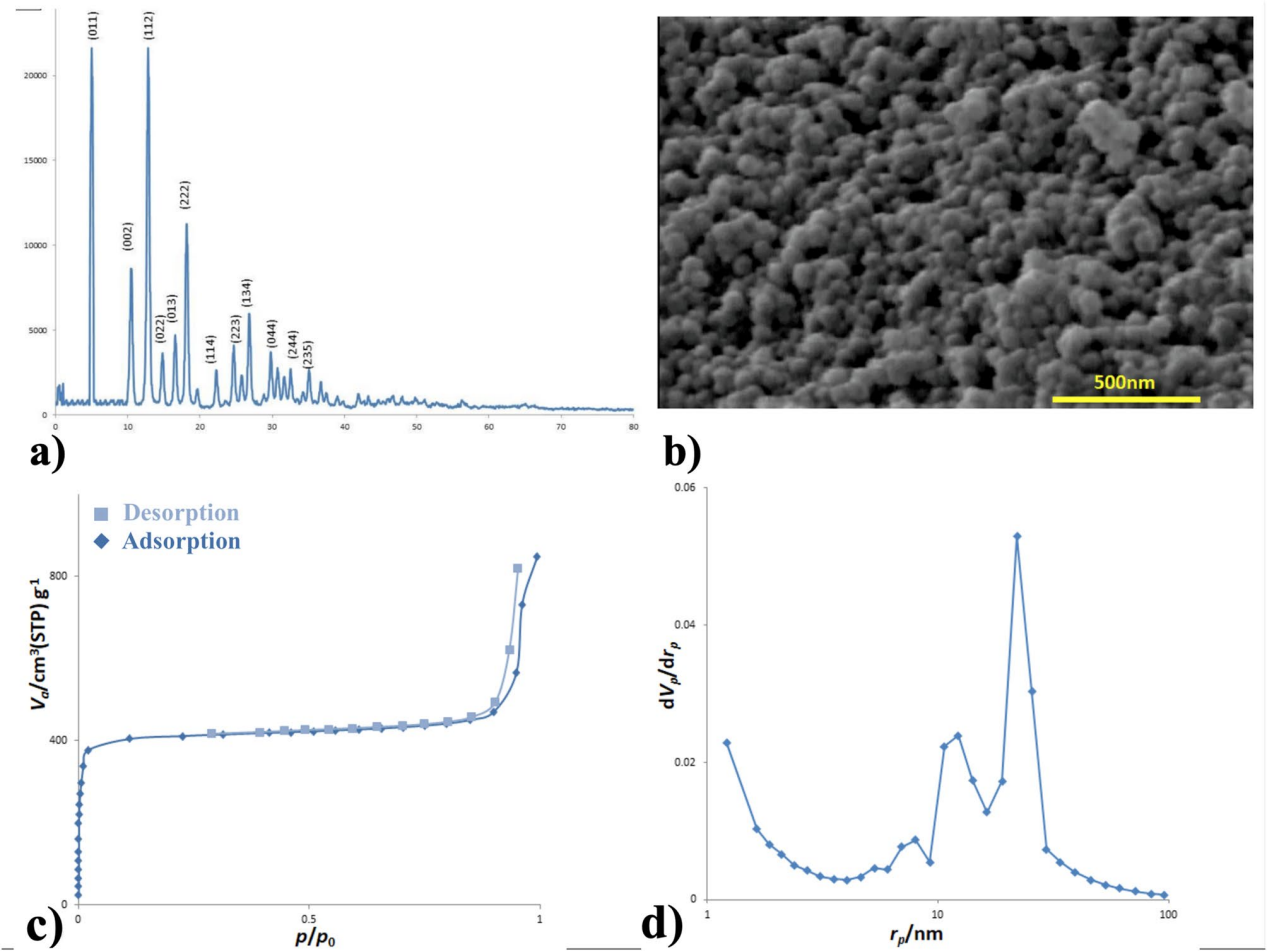
Chracteristics of synthesized MOF particles. (**a**) XRD pattern. (**b**) SEM image. (**c**) Nitrogen absorption/desorption isotherms. (**d**) BJH pore-size.

Different analytical techniques were employed to determine the morphology, porosity, and structure of the synthesized ZIF-8 MOFs. Table 2 summarizes the results. Figure 1 shows the characteristics of the ZIF-8 MOFs. The XRD pattern indicates well-defined crystal planes with slightly broadened peaks (Fig. 1a). In agreement with the simulated pattern of the ZIF-8 structure^39^, the particles are crystallized in a hexagonal structure^40^. The SEM image indicates that the particles have thin plate-like morphology with a lateral size of 70 to 80 nm and thickness of about 22 nm, yielding an aspect ratio of 0.3 (Fig. 1b). BET analysis at 77 K determines a type I adsorption isotherm with a small hysteresis loop (Fig. 1c). Analysis of the pore size distribution (Fig. 1d) reveals the nanoporous structure of MOF with the characteristics reported in Table 2. As seen, the processed MOF nanoplates have a very high specific surface area (1789 m^2^ g^−1^) and large pore volume (1.28 cm^3^ g^−1^).

**Table 2:** BET data for ZIF-8 MOFs prepared by solvothermal method.

Particle size (nm)	Specific surface area (m^2^/g)	Average pore diameter (nm)	Total pore volume (cm^3^/g)
65 ± 5	1789	2.9	1.29

FTIR spectroscopy exhibits characteristic peaks at 993 cm^−1^, 1142 cm^−1^, and 1421 cm^−1^, which are associated with the C–N stretching vibration^41^. Two small peaks at 3130 cm^−1^ and 2927 cm^−1^ are ascribed to aromatic C–H stretching vibration and aliphatic C–H stretching vibration of the imidazole ring and the methyl group, respectively^42^. The peak at 1583 cm^−1^ is attributed to C=N stretching vibration. Other peaks that appeared below 1250 cm^−1^ correspond to the in-plane and out-of-plane bending of the imidazole ring^43^. Zn–N stretching vibration is detected at 422 cm^−1^. The peaks in the range of 1350 to 900 cm^−1^ could be attributed to in-plane bending of the ring while the peaks at 754 and 688 cm^−1^ are associated with aromatic sp^2^ C–H bending^41^.

### Characteristics of the fibrous composite coating

**Figure 2:**
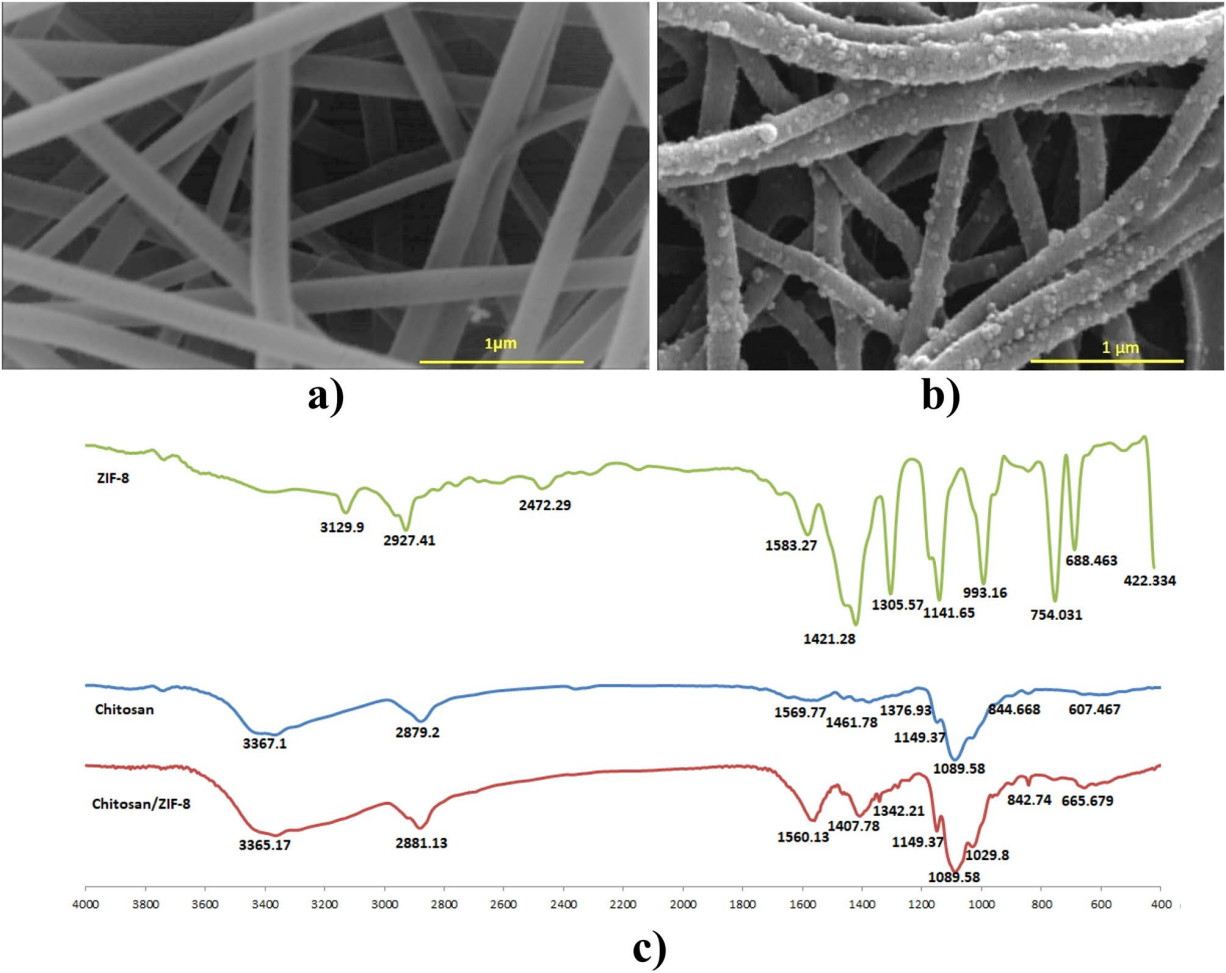
Characteristics of electrospun films formed on the surface of AZ91 magnesium alloy. Top-view SEM images of (**a**) chitosan and (**b**) MOF/chitosan coatings. (**c**) FTIR spectrum of chitosan, ZIF-8, and MOF/chitosan composite films.

Figure 2 shows the characteristics of chitosan and MOF/chitosan films deposited on the surface of AZ91 magnesium alloy by electrospinning. Top-view SEM images exhibit the fibrous structure of the coatings (Fig. 2a,b). Both films consist of uniform and fine fibers with an average diameter of 215 nm (for chitosan) and 200 nm (for MOF/chitosan composite). The fine particles decorated the surface of the nanofibers with a slight reduction in the average fiber diameter. The FTIR spectrum of chitosan (Fig. 2c) exhibits characteristic peaks at 3367 cm^−1^, 2879 cm^−1^, and 1570 cm^−1^ corresponding to O–H stretching, C–H symmetric and asymmetric stretching, and NH bending of the primary amine, respectively^42^. Notably, bands at 1645 (CO stretching of amide I) and 1550 (NH bending of amid II) are not visible, probably due to overlapping with others^44^. The CH_2_ bending and CH_3_ symmetrical deformations (C–O stretching vibration of CH_2_OH groups) are detected at around 1462 cm^−1^ and 1377 cm^−1^, respectively. The absorption bands at 1149 cm^−1^ and 1090 cm^−1^ can be attributed to asymmetric stretching of the C–O–C bridge and C–O stretching, respectively. The small bands at 608 cm^−1^ correspond to the wagging of the CS saccharide structure^42^. No major difference or band shifts is noticeable in the FTIR spectrum of MOF/chitosan film (Fig. 2c), indicating that chemical reactions or new band formation have not occurred upon processing.

### Biodegradation of magnesium alloy

**Figure 3:**
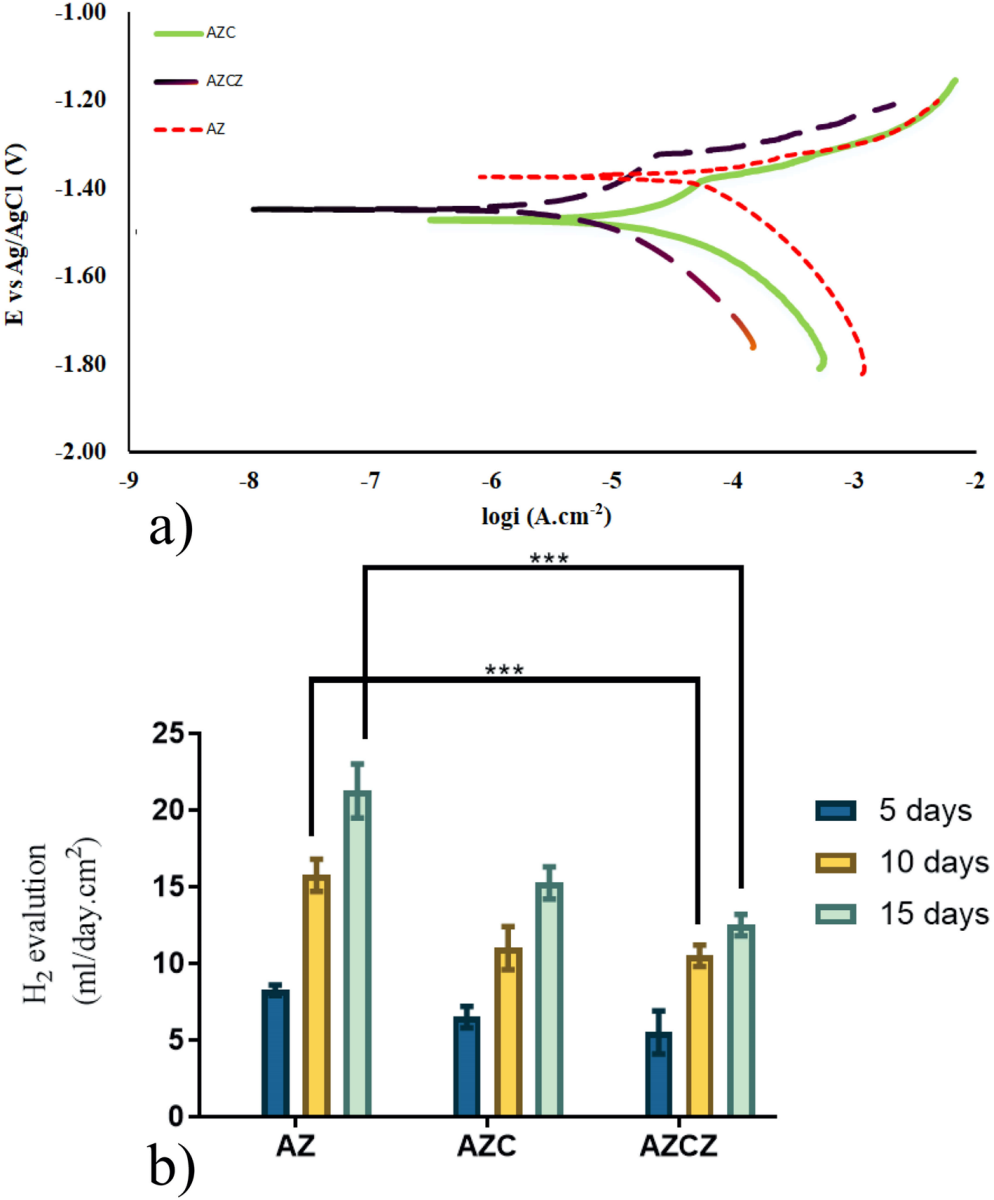
Electrochemical evaluations of the biodegradation of AZ91 alloy in SBF at 37 °C. (**a**) Tafel plots showing the effect of surface modification on the corrosion rate. (**b**) The average rate of hydrogen gas evolution at different time intervals.

To study the biodegradation of AZ91 alloy with and without surface modification, the electrochemical responses of the Specimens by triplicate of AZ, AZC, and AZCZ samples in SBF were measured and then the Tafel diagram was drawn for each of the samples and the values obtained were averaged and reported in Table 3. Also, the degradation rate of samples was calculated using the mpy formula. The Tafel plots of the examined specimens are shown in Fig. 3a. The extracted data from the polarization curves are summarized in Table 3. The results indicate that the magnesium alloy exhibits a relatively high corrosion current (*i*_*corr*_ = 34.2 µA cm^-2^) in SBF, which is comparable with other studies such as Mena-Morcillo and Velleva^36^, Zheng et al.^45^, and Razavi et al.^46^. Surface modification by the chitosan film significantly reduces the degradation rate and corrosion current to about half (*i*_*corr*_ = 16.6 µA cm^−2^). The potential (E_corr_) also slightly increases. Interestingly, the incorporation of ZIF-8 MOFs in chitosan further decreases the degradation rate and corrosion current by ⁓ 65% without changing *E*_*corr*_. As compared to unmodified magnesium alloy, *i*_*corr*_ drops to 6.5 µA cm^−2^, which is only about 20% of that of AZ91. Herein, it is important to mention that the electrochemical results on Mg alloys highly vary with the chemical composition, the type of the electrolyte, the surface condition, and the testing condition^47–49^. Therefore, it is difficult to compare directly our results with other studies. Employing a dense and bioinert layer on the magnesium surface yields lower degradation rates but this method suffers from good cell attachment and bioactivity. Porous and active layers such as fibrous membranes provide a better platform for bone integration although the degradation rate is higher.

**Table 3:** Effect of surface modification on the degradation rate of AZ91 alloy in SBF at 37 °C.

Coating	E_corr_ (V)	i_corr_ (µA/cm^2^)	β_a_ (mV/decay)	β_c_ (mV/decay)	Degradation rate (mm/year)
AZ	− 1.42	34.2 ± 0.45	69.1	272.7	29.9
AZC	− 1.47	16.6 ± 0.28	161.2	113.5	14.5
AZCZ	− 1.45	6.5 ± 0.11	223.5	169.1	5.6

The amount of hydrogen evolution measured at different time intervals (up to 15 days) after immersion in SBF is shown in Fig. 3b. The amount of gas evolution increases with increasing the immersion time for all specimens (Table 4). The rate of gas evolution exhibits a declining trend with time, indicating the effect of degradation products on the corrosion rate. It seems that the products of degradation act as a physical barrier to prevent rapid degradation in PE.

**Table 4:** The calculated corrosion rates (mm/year) at different time intervals based on the hydrogen evolution data.

Specimen	5 days	10 days	15 days
AZ	1.815	3.465	4.62
AZC	1.43	2.42	3.35
AZCZ	1.21	2.31	2.75

**Figure 4:**
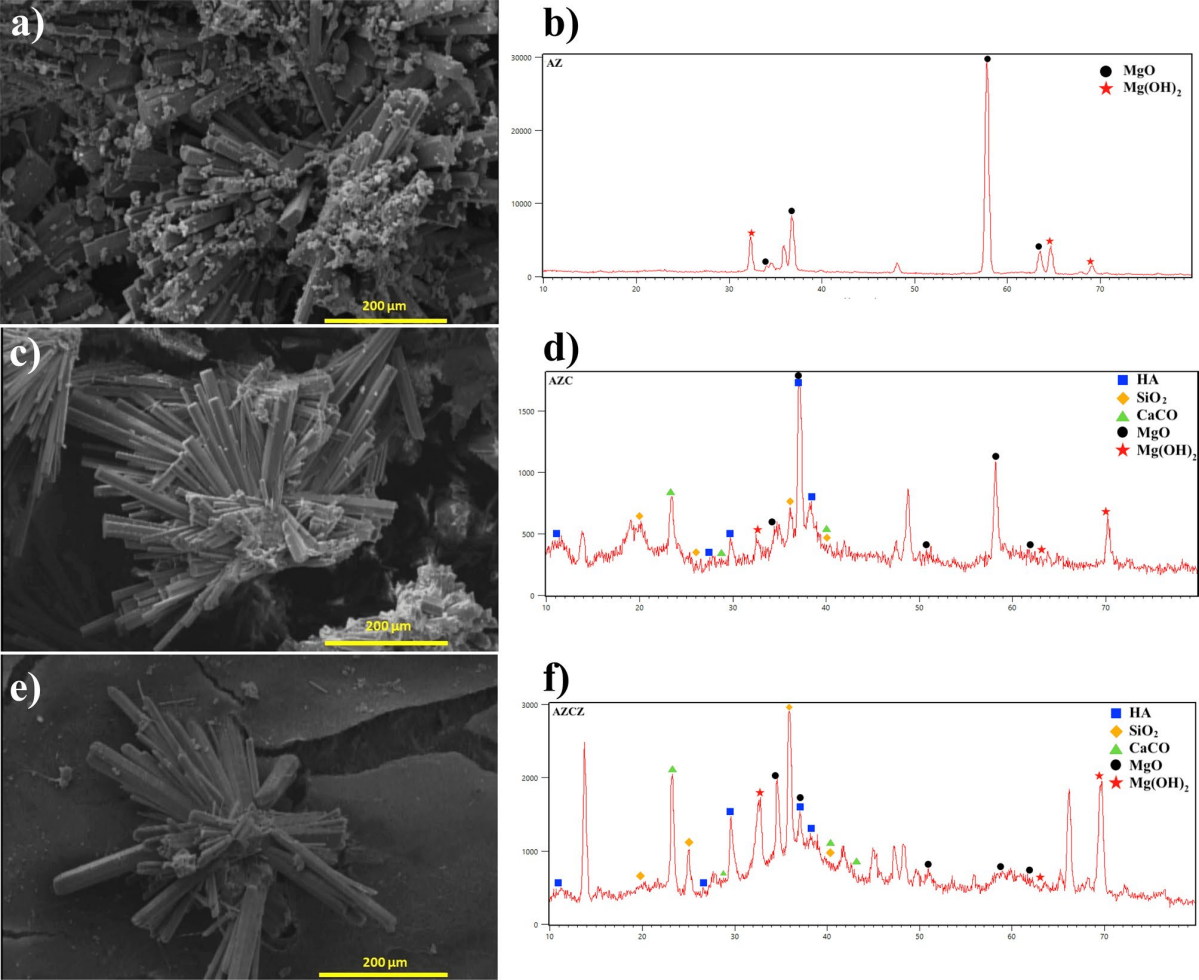
Products of biodegradation formed the surface of magnesium alloy in SBF at 37 °C after 15 days. SEM images and XRD patterns are for (**a**,**b**) AZ, (**c**,**d**) AZC, and (**e**,**f**) AZCZ samples.

It is known that magnesium hydroxide (Mg (OH)_2_) and magnesium oxide (MgO) are commonly formed during degradation and form a passive layer on the metal surface^50^. To study the degradation products, SEM and XRD were employed (Fig. 4). SEM studies show the formation of rod-shaped or flower-like precipitates on the surface. The XRD pattern of unmodified Mg alloy determines that basically magnesium hydroxide and magnesium oxide phases are formed. Small amounts of calcium carbonate and silicon oxide are also detected. The degradation products of the chitosan film are mostly hydroxyapatite. On the MOF-containing film, more complex phases are formed, owing to the presence of Zn ions. Besides, small amounts of Na_3_MgC_2_O_6_ and Mg (HCO_3_) (OH)_2_ (H_2_O) are detected. Anyway, the results of hydrogen evolution determine that the surface modification of the magnesium alloy by chitosan film significantly reduces the gas release by 30%. The incorporation of ZIF-8 MOF nanoplates further decreases the gas evolution by 40%.

### Electrochemical impedance spectroscopy

**Figure 5:**
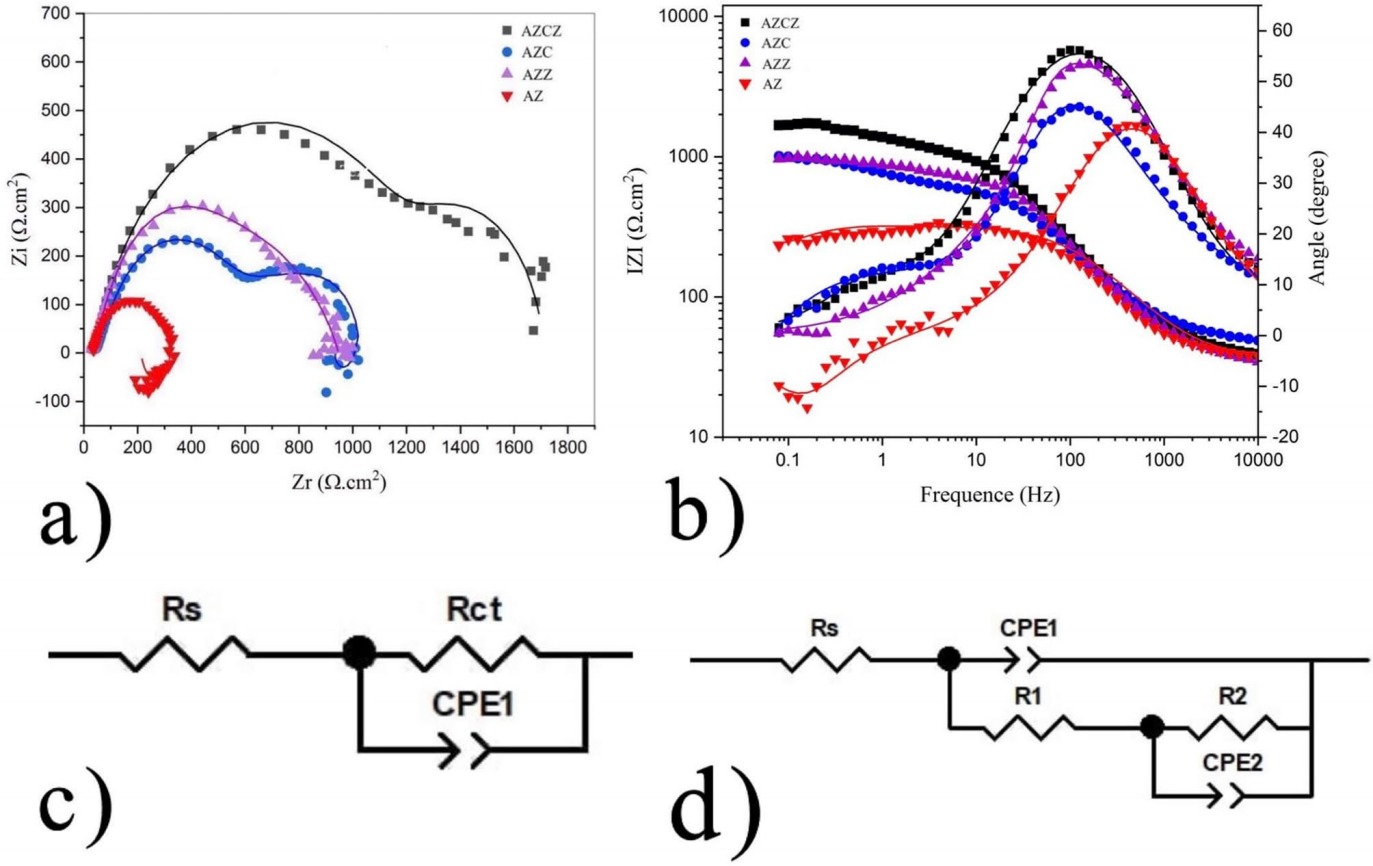
Electrochemical impedance spectroscopy of the biodegradation process of AZ91 in SBF. Effect of surface modification by chitosan and chitosan-MOF films on the (**a**) Nyquist and (**b**) Bode plots. The simulated equivalent circuits for (**c**) bare and chitosan/MOF modified alloy, and (**d**) chitosan-modified specimen. The fitting parameters are reported in Table 5.

To explore the mechanism of the biodegradation retardant effect of chitosan and MOF nanoplates, electrochemical impedance spectroscopy (EIS) in SBF at 37 °C was employed. The resulting Nyquist and Bode plots are shown in Fig. 5. In the Nyquist plots, two capacitive loops in both high and low-frequency regions are observed (Fig. 5a). In concurrence, the phase angle Bode plots exhibit two-time constants (Fig. 5b). After immersing the specimens in the PE solution, the biodegradation products are formed on the surface and two capacitive loops (at high- and low- frequency regions) develop due to changes in the charge transport, surface chemistry, and mass transfer^51^. The high-frequency region of the EIS relayed on the electrolyte penetration process including water uptake and the electrolyte interference. The diameter of the loops is directly proportional to the surface film resistance for mass and charge transport^52,53^. The unmodified magnesium alloy exhibits relatively a small loop that indicates a rapid biodegradation rate. The surface modification by chitosan film increases the diameter of the circle with double humps. The behavior of the specimen can be described based on capacitive behavior originated from the degradation of the magnesium alloy in the medium and inductive behavior resulted from continuous adsorption and desorption chemicals elements or ions and formation of corrosion products^37^. The incorporation of ZIF-8 MOFs further enlarges the circles with little effect on the inductive behavior. Therefore, the chitosan-MOF film has further increased the degradation resistance of the magnesium substrate.

To get a better insight into the mechanism of biodegradation, the equivalent circuit for the Nyquist plot was constructed (Fig. 5c,d). The values obtained by data fitting are reported in Table 5. As so the low-frequency region for the bare and chitosan-MOF specimens is too much noisy, an equivalent circuit with a one-time constant was used to fit the EIS plots. However, a circuit with two-time constants was used to fit the chitosan-modified AZ91 sample. R_s_ is representative of the solution resistance which is in series with the unit of the oxide layer system. For the bare electrode, R1 and R_2_ in parallel with constant phase elements (CPE_1_ and CPE_2_) are the resistance of naturally formed oxide film on the substrate and charge transfer resistance of the faradaic process on the metal surface, respectively^52,53^. For the chitosan and chitosan-MOF specimens, the resistances (R_1_ and R_2_) and constant phase elements (CPE_1_ and CPE_2_) represent the resistance of the porous outer layer and the inner coating/substrate interface, respectively. This part of the circuits was not quantitatively evaluated for the bare and chitosan-MOF coated AZ91 electrodes as the corresponding error was relatively high. Noted that the value of fitting errors should be less than 1%^54,55^. Besides, CPE which models the behavior of a double layer (as an imperfect capacitor) was considered instead of a capacitive element because of the surface inhomogeneity and possible diffusional factors affecting the shape of the semi-circles^56^. Table 5 indicates that the R_2_ value for the chitosan-MOF specimen (1256 Ω) is much higher than that of the chitosan-modified AZ91 (651.9 Ω) and the bare sample (291.3 Ω). This finding affirms corrosion resistance enhancement of AZ91 through surface modification by the chitosan-MOF film via inserting a physical barrier against diffusion/penetration of the corrosive ions from the solution toward the metal surface. For the chitosan-modified AZ91 specimen, the value of R2 (651.9 Ω) is far higher than that of R_1_ (10.6 Ω). This observation could indicate the vital role of the magnesium/coating interface in the biodegradation process.

**Table 5:** Fitting parameters of the equivalent circuit constructed based on EIS results.

Coating	R_s_ (Ω cm^2^)	R_1_ (Ω cm^2^)	R_2_ (Ω cm^2^)	CPE_1_ (μF. cm^2^)	CPE_2_ (μF cm^2^)
AZ	35.5	–	291.3	–	24.9
ACZ	47.7	10.6	651.9	800	30.6
AZCZ	38.5	–	1256	–	20.1

### In vitro biocompatibility and cell adhesion

**Figure 6:**
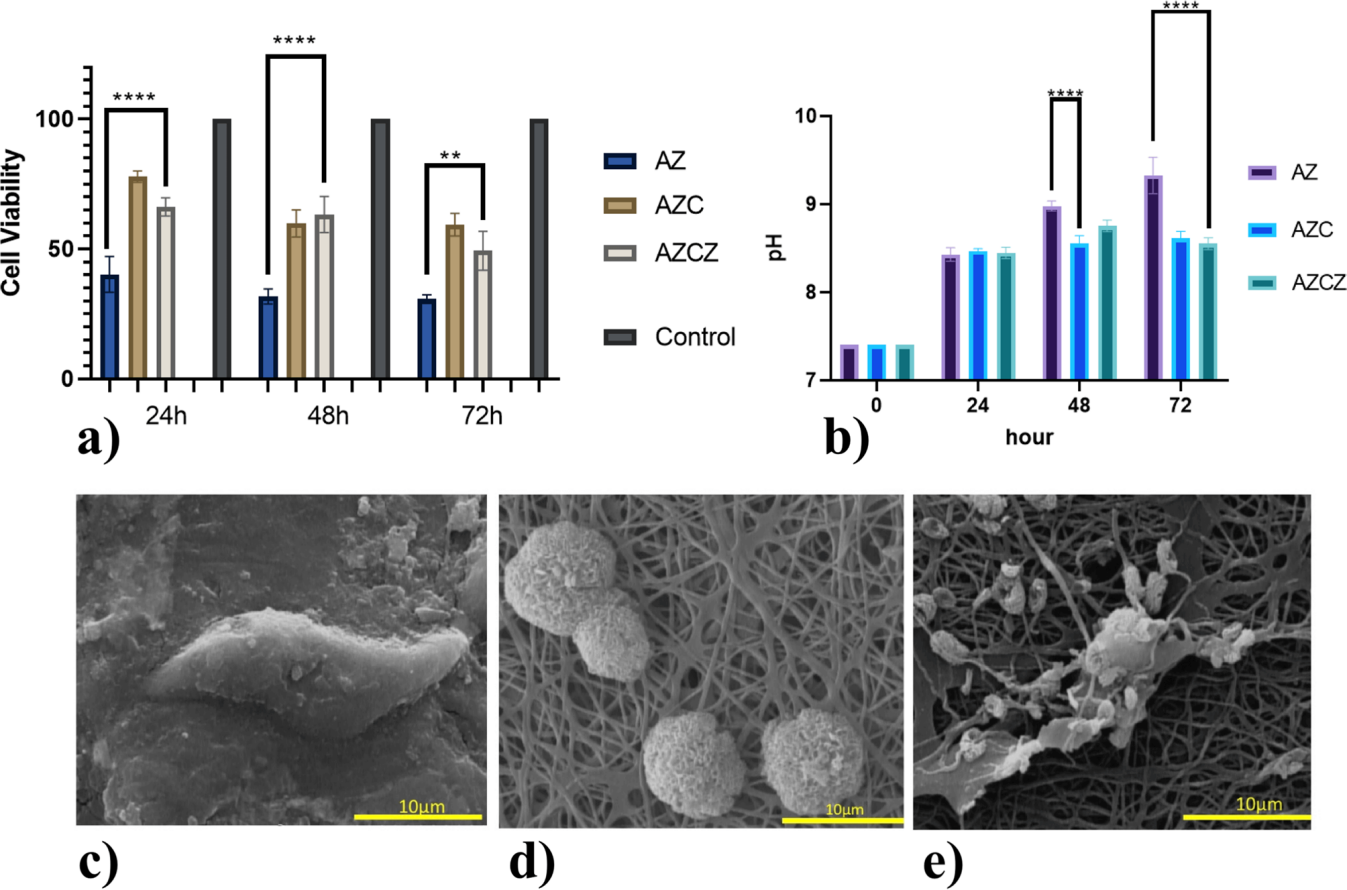
Effect of surface modification on the in vitro biocompatibility of AZ91 alloy. (**a**) MTT assay using fibroblast cells. (**b**) pH variation of AZ91 based implants after incubation in cell media. SEM images showing the adhesion and spreading of MG63 cells on the surface of (**c**) AZ, (**d**) AZC (**e**) AZCZ.

To assay the in vitro biocompatibility of the specimens, L929 cells were used for MTT assay (Fig. 6a). The unmodified AZ91 alloy exhibits poor biocompatibility with cell viability of less than 50% even after a short incubation time (24 h). A longer incubation time further decreases the cell viability, indicating the cytotoxic effect of Mg degradation, as reported in the literature^57^. The magnesium alloy modified with chitosan film exhibits higher cell viability (> 90%) at the early stage but reduced to about 70% at prolonger times. Moderate biocompatibility is attained for the chitosan-MOF film without a major effect of the incubation time (within the examined period). Nevertheless, the cell viability is at least double of the unmodified Mg alloy. The attachment of MG63 cells on the surface of the specimens incubated for 72 h is shown in Fig. 6c–e. No live cells are observable on the surface of unmodified Mg alloy due to the rapid degradation and formation of corrosion products. In contrast, the cells have adhered to the fibrous chitosan film but the formation of filopodia and spreading is not noticeable. The chitosan-MOF film results in the formation of relatively better cell adhesion and spreading.

## Discussion

In this study, ZIF-8 nanoplates were employed and incorporated in the chitosan nanofiber to modify the biodegradation rate of AZ91 Mg alloy in PE. It was shown that the MOF particles decorated the surface of the nanofibers and slightly decreased their diameters. The reduced sizes can be attributed to the increase in the size of the Taylor cone and decrease in the jet velocity due to a change in the viscosity of the solution^58,59^. Since ZIF MOFs are hydrophilic^39^, the particles tend to be pushed toward the outer layer and collected on the surface. Meanwhile, no remarkable agglomeration was noticed.

The results indicated that the chitosan coating containing MOFs exhibited better protection against biodegradation in PE. The advantage of using ZIF-8 is ascribed to its anti-corrosion properties^60,61^ that can reduce the electrochemical potential and current density of AZ91 alloy^24,62^ as well as its high stability in physiological environment and drug loading capacity^24–28^. Although our coating materials seem to be less protective as compared to bioinert and dense protective layers^23,63–65^, employing fibrous membranes provide a better platform for cell adhesion and bone integration.

Electrochemical studies revealed that the coatings were mainly operated as a physical barrier. EIS studies determined that the impedance of the surface-modified specimens was higher than that of the bare metal. It seemed that the coating layer reduced the ion diffusion and thus decreased the corrosion rate^37^. The Bode plot determined low-frequency and high-frequency resistor zones and a capacitive behavior in the mid-frequency range due to the maximum phase changes (θ_max_) vs. log(f)^66^. Based on the shape and values of θ at high frequencies, it appeared that the electrochemical response of the surface-modified samples had a higher tendency toward capacitive behavior. At low frequencies, due to absorption and reduction of corrosion products such as Mg (OH) and Mg (OH)_2_ at the interface between the electrolyte solution and the metal surface, an induction loop appears that is equivalent in shape to the inductor and offers resistance^67^. The Nyquist plots and the capacitive loops also indicated the existence of a distinct layer on the surface of the specimens. The diameter of the capacitive ring indicated the higher polarization resistance of the chitosan-MOF composite specimen^68^. Here, the role of the precipitates on the corrosion rate should also be considered, It was shown that corrosion of magnesium in PE was accompanied by the formation of precipitates on the surface (Fig. 4). Degradation and dissolution of magnesium increased the pH of the medium that promoted phosphate formation due to saturating of SBF at highly alkaline conditions^50^. The rise of the pH is attributed to the biodegradation of the metal surface and the release of hydroxide ions in the cell culture medium^33,50^ as well as the formation of hydrogen bubbles^10^. The surface modification reduced the amount of pH raise as the coating layer acted as a barrier against direct contact of the medium and the metal surface. Not only the fast release of Mg in the culture medium affects the cell viability due to the alkalization, but also the degradation of MOFs may influence the cell response.

The cell viability assay indicated improved biocompatibility of chitosan-modified specimens over 72 h. Measurements of the pH value of the culture medium (Fig. 6b) indicated that for the bare metal, the pH exceeded beyond the physiological level and gained a high value (8.6) that cells can tolerate. Hook et al.^24^ showed that at concentrations beyond 30 µg mg^−1^, ZIF-8 exhibited cytotoxicity due to the release of Zn2^+^ in mitochondrial ROS products. This negative effect suppresses the cell cycle in the G2/M phase, which is irreversible and finally caused DNA damage and ultimately activates cellular apoptosis pathways^34^. We used a low amount of ZIF-8 in our mats; therefore, the effect of Zn ions should be marginal. It was also shown that cell adhesion on the surface of the fibrous coatings was improved. Cells usually interact better with surfaces that have more pores and roughness^69^. Hydrophilicity and mechanical durability of the surface also affect the interaction of cells with the material at the interface^30,57^. For instance, it has been shown that MG63 osteoblast-like cells significantly proliferate at rough surfaces such as acid-etched or plasma^69,70^. Hydrophilic surfaces have much more integration and adhesion with osteoblastic cells with implants compared to hydrophobic or less hydrophilic surfaces^57^.

## Conclusions

To control the biodegradation rate of AZ91 magnesium alloy in physiological environments, composite films of ZIF-8 MOF/chitosan were deposited on the surface by electrospinning. Plate-like MOF particles with lateral sizes of 70–80 nm with an aspect ratio of 0.3 and a specific surface area of 1789 m^2^ g^−1^ were prepared by solvothermal methods. Continuous, uniform, and bead-free fibers with an average diameter of 30 µm were deposited on the surface of the magnesium alloy. The incorporation of ZIF-8 particles in the chitosan coating did not significantly change the size and uniformity of the fibers, but the particles tended to decorate the surface of the polymer. In vitro evaluations of biodegradation in PE by electrochemical methods indicated that the corrosion current of AZ91 alloy decreased by ⁓ 50% after surface modification by the fibrous chitosan coating. The incorporation of MOF particles further reduced the corrosion current by ⁓ 65%. The rate of hydrogen evolution was also reduced significantly. EIS determined that the polymer and composite films operated as a physical barrier and increased the charge transfer resistance while changing the capacitive behavior of the biodegradation process. The degradation products were changed from magnesium compounds for the bare metal to hydroxyapatite precipitates with more complex Ca–Zn–Si compounds. In vitro cytotoxicity examinations also revealed that surface modifications improved the biocompatibility due to the reduced degradation rate of Mg in the physiological medium. In contrast to the bare metal, cell adhesion with filopodia formation and cell spreading were also improved. Therefore, the MOF-chitosan composite coating showed promising potential for the surface modification of magnesium alloys to limit their biodegradation in vitro. In vivo examinations, however, are required to evaluate the biological response of the surface-modified BMIs to reveal their potential applications for bone regeneration.
